# Validity of regional network systems on reperfusion therapy in diabetes mellitus and non-diabetes mellitus patients with ST-segment elevation myocardial infarction

**DOI:** 10.3389/fcvm.2022.991479

**Published:** 2022-11-25

**Authors:** Xicong Li, Lifei Lu, Qi Yuan, Lixia Yang, Liping Du, Ruiwei Guo

**Affiliations:** ^1^Department of Cardiology, Kunming Medical University, The 920th Hospital, Kunming, Yunnan, China; ^2^State Key Laboratory of Respiratory Disease, National Clinical Research Center for Respiratory Disease, National Center for Respiratory Medicine, Guangzhou Institute of Respiratory Health, The First Affiliated Hospital of Guangzhou Medical University, Guangzhou, China; ^3^Department of Cardiology, 920th Hospital of Joint Logistics Support Force, People’s Liberation Army of China (PLA), Kunming, Yunnan, China; ^4^Proctology Department of Traditional Chinese Medicine, First People’s Hospital of Yunnan Province, Kunming, China

**Keywords:** ST-segment elevation myocardial infarction, reperfusion therapy, regional network systems, diabetes mellitus, FMC to wire

## Abstract

**Background:**

Patients with ST-segment elevation myocardial infarction (STEMI) with diabetes mellitus (DM) had higher mortality and poorer prognosis than those without DM. Previous studies had demonstrated the effectiveness of regional network systems (RNS) for reperfusion therapy in patients with STEMI. However, the differences in nursing care with RNS in subgroups of patients with DM with STEMI were unclear. Our study aimed to evaluate the validity of RNS in reperfusion therapy in patients with STEMI with or without DM.

**Methods:**

We retrospectively enrolled patients with STEMI who received reperfusion therapy at the chest pain center of the 920th Hospital in Kunming City, Yunnan Province from 2019 to 2021. Personal information and hospitalization information for patients with STEMI were collected through the chest pain center registration system. Univariate and multivariate logistic regression were used to analyze factors associated with outcomes in patients with STEMI who received RNS. Wilcoxon rank-sum test and chi-squared test were used to analyze the differences in reperfusion therapy times and clinical outcomes between RNS and non-RNS in patients with STEMI with or without DM.

**Results:**

This study enrolled 1,054 patients with STEMI, including 148 patients with DM and 906 patients without DM. Logistic regression analysis indicated that DM was associated with patients with STEMI who received RNS [OR 1.590 95% CI (1.034–2.446), *P* = 0.035]. RNS may decrease the reperfusion therapy time in patients with STEMI and patients without DM with STEMI, including the first medical contact (FMC) to door, FMC to wire and FMC to catheterization laboratory activity (all *P* < 0.05). However, we found no significant difference in reperfusion therapy times with and without RNS in patients with DM (all *P* > 0.05).

**Conclusion:**

Regional network systems may decrease the reperfusion therapy time in patients without DM with STEMI, but no decrease was found in patients with DM with STEMI.

## Introduction

Diabetes mellitus (DM) was an important and common chronic disease, which can cause metabolic disorders in patients, thus resulting in vascular inflammation, endothelial dysfunction and thrombosis (1). These mechanisms may be closely associated with an elevated risk of coronary heart disease and poor prognosis. Acute heart failure or cardiogenic shock accounted for more than 80% of the mortality in hospitalize patients with DM (2). Previous studies had reported that hospital mortality rates among patients with DM with ST-segment elevation myocardial infarction (STEMI) were more than twice those of patients without DM in men and were even higher in women (2–4). Compared with patients without DM, patients with DM had higher short-term and long-term mortality rates after heart attack (5). Therefore, these patients required better medical treatment to improve their condition.

A regional network system (RNS) in the United States, involving regional collaborative treatment programs based in hospitals able to provide percutaneous coronary intervention (PCI), as implemented by state or interstate units, had been independently associated with shorter admission times (6). Previous studies had found that RNS use can decrease the reperfusion therapy time in patients with STEMI (7–9). However, patients with STEMI and DM had poorer prognosis than those without DM, and the extent to which RNS might decrease the time of reperfusion therapy in patients with DM with STEMI was unclear.

The purposes of our study were (1) to retrospectively analyze whether RNS might improve the reperfusion therapy time for patients with STEMI with or without DM, and (2) to analyze factors associated with STEMI in patients with DM to understand and improve the treatment plans for these patients.

## Materials and methods

### Regional network system

The 920th Hospital is located in Kunming, an important central city in western China. Since its cardiology department first opened a rapid diagnosis and treatment channel for interventional diagnosis and treatment of AMI in Yunnan Province in 2003, the chest pain center (CPC) has been staffed by cardiology, emergency and ambulance pre-hospital first aid personnel in the core, joint radiology, laboratory and other related departments, and currently comprises 24 non-PCI network hospitals at the grass-roots level. A set of effective clinical pathways and a diagnostic operation flow were established; for example, a WeChat group between ambulances and network hospitals has been used to achieve timely transmission of pre-hospital electrocardiography (ECG) results and patient information to the center, thus providing a seamless connection between the green channel in the hospital and the pre-hospital first aid system. The preoperative preparation and catheterization can thus be completed before patients with STEMI and high-risk non-STEMI arrive at the hospital with emergency PCI indications. Patients can arrive directly at the catheter room, bypassing the emergency department and coronary care unit, thus greatly shortening the time before reperfusion treatment.

### Study design

We retrospectively enrolled patients with STEMI who received reperfusion therapy at the CPC in the 920 Hospital in Kunming city between 2019 and 2021. Personal information and hospitalization information for patients with STEMI were collected through the CPC registration system, including age, sex, smoking history, DM history, admission status, cardiac function grade, complications (hypertension, hyperlipidemia, obesity, and family history of CVD, or coronary heart disease), remote ECG, thrombolytic therapy, clinical outcomes (hospitalization cost and time and discharge status) and reperfusion therapy time, including the first medical contact (FMC) to door (10), FMC to wire (11) and FMC to catheterization laboratory activity (CLA).

Patients with the following conditions were excluded: (1) AMI occurring in our hospital; (2) chest pain without symptoms or hemodynamic disorder lasting more than 12 h; (3) contraindications for antiplatelet or anticoagulation, such as active peptic ulcer, thrombocytopenia, severe coagulation dysfunction or hemorrhagic disease; (4) severe valvular heart disease, cardiomyopathy, severe infection or severe hepatorenal insufficiency; (5) allergy to contrast agents or stent materials; (6) refusal (of patients or family members) to accept emergency interventional examination; (7) missing information and data on diagnosis and treatment records. (8) Patients in the present study had a first STEMI and admitted within 12 h after symptom onset (FMC-door ≤ 12 h) (12, 13). The diagnostic criteria for type 2 DM were typical diabetes symptoms (polydipsia, polyuria, polyphagia or unexplained weight loss) and random venous plasma glucose ≥ 11.1 mmol/L, fasting venous plasma glucose ≥ 7.0 mmol/L or plasma glucose ≥ 11.1 mmol/L 2 h after an oral glucose tolerance test (14). AMI should be used when evidence of myocardial injury (defined as an elevation of cardiac troponin values with at least one value above the 99th percentile upper reference limit) is observed with necrosis in a clinical setting, findings are consistent with myocardial ischemia, or persistent chest discomfort or other symptoms indicating ischemia and ST segment elevation are observed in at least two adjacent leads—findings usually resulting in STEMI diagnosis (15, 16). We further divided the DM group and the non-DM group into an RNS group and No RNS group.

All patients provided informed consent for study participation, and the research study was approved by the Medical Ethics Committee of the 920 Hospital of Kunming Medical University (2015067).

### Statistical analysis

Statistical analysis was performed in SPSS statistics version 26.0 (IBM Corp. Armonk, NY, USA). Continuous variables showing a normal distribution are presented as mean ± standard deviation, and continuous variables without a normal distribution are presented as median [interquartile range (IQR)]. We compared baseline characteristics between the RNS group and non-RNS group with Student’s *t*-test, Wilcoxon rank-sum test or chi-squared test. Univariate and multivariate logistic regression was used to analyze factors associated with outcomes in patients with STEMI who received RNS treatment. Wilcoxon rank-sum test and chi-squared test were used to analyze the differences in reperfusion therapy times and clinical outcomes in patients with STEMI with or without DM with RNS. All reported *P*-values were two-sided. We assumed significance at the 5% level (*P* < 0.05).

## Results

### Baseline characteristics

**FIGURE 1:**
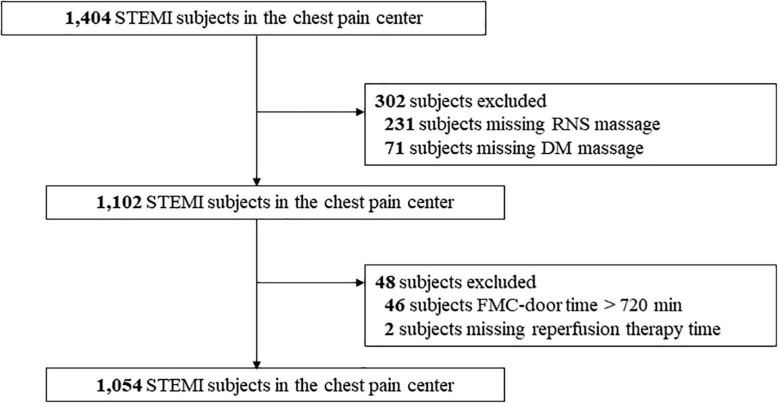
Flow chart of selection process and dropouts of the present study. FMC-door, the first medical contact to door. RNS, regional network systems; DM, diabetes mellitus; STEMI, ST-segment elevation myocardial infarction.

A flow chart of the selection process and dropouts in the present study was showed in Figure 1. A total of 1,054 patients with STEMI were enrolled, including 148 (14.0%) patients with diabetes and 906 (86.0%) patients without diabetes. Table 1 shows the baseline characteristics of patients with and without DM. Compared with patients without DM, the patients with DM included more women (36.5 vs. 23.3%, *P* = 0.001), and had greater hypertension (58.8 vs. 39.7%, *P* < 0.001) and less hyperlipidemia (35.8 vs. 45.8%, *P* = 0.023). A total of 114 (77.0%) patients with DM received RNS treatment, whereas 34 (23.0%) patients with DM did not. No significant differences were observed in most variables between the RNS and No RNS groups in both patients with and without DM. The RNS group, compared with the No RNS group, had less hypertension (38.3 vs. 47.3%, *P* = 0.042), and more patients received remote ECG (98.7 vs. 93.2%, *P* < 0.001) among patients with DM.

**TABLE 1:** Baseline characteristics.

Variable	No DM (*n* = 906)				DM (*n* = 148)				DM vs. no DM
			
	All subjects	RNS	No RNS	*P*-value*	All subjects	RNS	No RNS	*P*-value*	*P*-value
Number, n (%)	906	760 (83.9)	146 (16.1)	−	148	114 (77.0)	34 (23.0)	−	−
Age, years	61.42 ± 12.26	61.26 ± 12.23	62.25 ± 12.44	0.368	61.49 ± 12.96	61.18 ± 13.15	62.53 ± 12.43	0.595	0.950
Sex, n (%)				0.199				0.145	**0.001**
Female	211 (23.3)	183 (24.1)	28 (19.2)		54 (36.5)	38 (33.3)	16 (47.1)		
Male	695 (76.7)	577 (75.9)	118 (80.8)		94 (63.5)	76 (66.7)	18 (52.9)		
Hospital state, n (%)				**0.045**				**0**.**030**	0.679
Intermittent chest pain	67 (7.4)	49 (6.4)	18 (12.3)		14 (9.5)	8 (7.0)	6 (17.6)		
Persistent chest pain	832 (91.8)	705 (92.8)	127 (87.0)		133 (89.9)	106 (93.0)	27 (79.4)		
Symptoms relieved	7 (0.8)	6 (0.8)	1 (0.7)		1 (0.7)	0 (0)	1 (2.9)		
Killip classification, n (%)				0.166				0.072	0.765
Level I	298 (32.9)	261 (34.3)	37 (25.3)		49 (33.1)	43 (37.7)	6 (17.6)		
Level II	565 (62.4)	465 (61.2)	100 (68.5)		93 (62.8)	66 (57.9)	27 (79.4)		
Level III	36 (4.0)	28 (3.7)	8 (5.5)		6 (4.1)	5 (4.4)	1 (2.9)		
Level IV	7 (0.8)	6 (0.8)	1 (0.7)		0 (0)	0 (0)	0 (0)		
Current smoking, n (%)	29 (3.2)	25 (3.3)	4 (2.7)	0.929	6 (4.1)	6 (5.3)	0 (0)	0.337	0.591
Complication, n (%)									
Hypertension	360 (39.7)	291 (38.3)	69 (47.3)	**0.042**	87 (58.8)	70 (61.4)	17 (50.0)	0.236	<**0.001**
Hyperlipidemia	415 (45.8)	350 (46.1)	65 (44.5)	0.734	53 (35.8)	44 (38.6)	9 (26.5)	0.196	**0.023**
Obesity	10 (1.1)	9 (1.2)	1 (0.7)	0.923	4 (2.7)	3 (2.6)	1 (2.9)	0.923	0.115
Family history of CVD	7 (0.8)	7 (0.9)	0 (0)	0.606	0	0	0	−	0.602
Coronary artery disease	38 (4.2)	31 (4.1)	7 (4.8)	0.693	6 (4.1)	6 (5.3)	0 (0)	0.172	0.937
Remote electrocardiogram, n (%)	886 (97.8)	750 (98.7)	136 (93.2)	**<0.001**	145 (98.0)	112 (98.2)	33 (97.1)	1.000	1.000
Thrombolytic therapy, n (%)	382 (42.2)	331 (43.6)	51 (34.9)	0.053	62 (41.9)	51 (44.7)	11 (32.4)	0.199	0.951

Data are presented as the mean ± standard deviation or median (interquartile range) and were analyzed with Student’s *t*-test or Wilcoxon’s rank-sum test.

DM, diabetes mellitus; No DM, without diabetes mellitus; CVD, Cardiovascular disease. *RNS, regional network systems; vs. No RNS, without regional network systems.

Bold values represent significant *p* values.

### Serological examination

Table 2 showed serological examination results for patients with STEMI with or without DM. Compared with patients without DM, patients with DM had higher triglyceride (TG) [1.30 (0.88–2.53) vs. 1.00 (0.69–1.49), *P* < 0.001]and lower high-density lipoprotein cholesterol (HDL-C) [1.15 (0.99–1.42) vs. 1.25 (1.05–1.50), *P* = 0.042]. Patients with STEMI who received RNS had lower troponin than those who did not and this result was observed in patients with or without DM. Meanwhile, we found that patients with STEMI who received RNS had lower TG [0.97 (0.66–1.42) vs. 1.20 (0.83–1.60), *P* = 0.024] in no-DM patients but no found in DM patients [1.36 (0.86–2.52) vs. 1.20 (1.07–3.00), *P* = 0.657].

**TABLE 2:** Serological examination between DM and no DM subjects.

Variable	No DM (*n* = 906)				DM (*n* = 148)				DM vs. no DM
			
	All subjects	RNS	No RNS	*P*-value*	All subjects	RNS	No RNS	*P*-value*	*P*-value
Troponin protein, μg/L	26.93 (20.04–27.00)	26.50 (20.30–27.00)	27.05 (14.80–61.38)	**0.001**	27.00 (24.00–30.76)	27.00 (23.39–27.00)	56.00 (26.49–101.20)	**0.004**	0.167
BNP, pg/mg	120.00 (61.20–237.75)	123.20 (56.00–244.00)	105.00 (70.45–229.00)	0.640	139.00 (82.40–303.00)	136.60 (63.70–300.50)	194.00 (106.00–372.25)	0.245	0.154
TC, mmol/L	4.86 (4.00–5.67)	4.99 (4.00–5.75)	4.56 (4.01–5.56)	0.436	4.80 (3.54–5.36)	4.90 (3.57–5.34)	4.34 (3.25–6.23)	0.822	0.439
TG, mmol/L	1.00 (0.69–1.49)	0.97 (0.66–1.42)	1.20 (0.83–1.60)	**0.024**	1.30 (0.88–2.53)	1.36 (0.86–2.52)	1.20 (1.07–3.00)	0.657	**< 0.001**
HDL-C, mmol/L	1.25 (1.05–1.50)	1.27 (1.05–1.50)	1.21 (1.05–1.60)	0.915	1.15 (0.99–1.42)	1.15 (1.00–1.41)	1.11 (0.95–1.81)	0.700	**0.042**
LDL-C, mmol/L	2.96 (2.30–3.64)	2.98 (2.33–3.64)	2.91 (2.18–3.64)	0.793	2.78 (1.92–3.37)	2.80 (2.48–3.37)	1.93 (1.30–3.67)	0.227	0.153

Data are presented as the mean (standard deviation) or median (interquartile range); serological examination parameters the maximum values at examination.

BNP, brain natriuretic peptide; HDL-C, high-density lipoprotein cholesterol; LDL-C, low-density lipoprotein cholesterol; DM, diabetes mellitus. No DM, without diabetes mellitus; RNS, regional network systems; No RNS, without regional network systems.

Bold values represent significant *p* values.

### Clinical outcomes

Table 3 showed the clinical outcomes of patients with STEMI with or without DM. Compared with patients without DM, patients with DM had worse adverse outcomes (death or transfer) (12.2 vs. 5.6%, *P* = 0.003). However, no significant difference was observed between patients with or without DM in hospitalization time [10.00 (8.00–12.00) vs. 10.00 (8.00–13.00), *P* = 0.171] and hospitalization costs [35.00 (27.27–46.40) vs. 35.00 (28.32–43.69), *P* = 0.846]. We found no statistically significant differences in hospitalization time, hospitalization costs and adverse outcomes between the RNS and No RNS groups in both patients with and without DM (all *P* > 0.05).

**TABLE 3:** Clinical outcomes between DM and no DM subjects.

Variable	No DM (*n* = 906)				DM (*n* = 148)				DM vs. no DM
			
	All subjects	RNS	No RNS	*P*-value*	All subjects	RNS	No RNS	*P*-value*	*P*-value
Hospitalization time, day	10.00 (8.00–13.00)	10.00 (8.00–12.00)	10.00 (8.00–13.00)	0.252	10.00 (8.00–12.00)	10.00 (7.75–12.00)	9.00 (8.00–11.25)	0.682	0.171
Hospitalization cost, Qian Yuan	35.00 (28.32–43.69)	35.00 (28.98–43.21)	35.00 (26.71–46.32)	0.545	35.00 (27.27–46.40)	35.00 (26.86–46.40)	35.00 (28.74–47.73)	0.794	0.846
Discharge condition, n (%)				0.138				1.000	**0.003**
Be cured or improved	855 (94.4)	721 (94.9)	134 (91.8)		130 (87.8)	100 (87.7)	30 (88.2)		
Death or transfer to hospital	51 (5.6)	39 (5.1)	12 (8.2)		18 (12.2)	14 (12.3)	4 (11.8)		

Data are presented as the mean (standard deviation) or median (interquartile range) and were analyzed with Student’s t-test or Wilcoxon’s rank-sum test.

DM, diabetes mellitus; discharge condition, the doctor assessed the patient’s condition before discharge. No DM, without diabetes mellitus; RNS, regional network systems; No RNS, without regional network systems.

Bold values represent significant *p* values.

### Relate factors for receiving regional network systems in ST-segment elevation myocardial infarction patients

Univariate logistic regression analysis indicated that hospital state [0.525, 95%CI (0.320–0.860), *P* = 0.011], Killip classification [1.476, 95%CI (1.123–1.940), *P* = 0.005] and DM [1.553, 95%CI (1.018–2.367), *P* = 0.041] were associated with receiving RNS. Multivariate logistic regression analysis indicated that hospital state [0.515, 95%CI (0.311–0.853), *P* = 0.010], Killip classification [1.425, 95%CI (1.072–1.893), *P* = 0.015] and DM [1.590, 95%CI (1.034–2.446), *P* = 0.035] were still associated with receiving RNS (Table 4).

**TABLE 4:** Relate factors for RNS.

Variable	Univariate OR (95%CI)	*P*-value	Multivariate OR (95% CI)	*P*-value
Sex (male vs. female)	1.076 (0.744–1.556)	0.698	1.207 (0.806–1.809)	0.362
Age	1.006 (0.993–1.019)	0.368	1.007 (0.993–1.021)	0.337
Current smoking	0.662 (0.257–1.704)	0.392	0.691 (0.238–2.006)	0.497
Hospital state	0.525 (0.320–0.860)	**0.011**	0.515 (0.311–0.853)	**0.010**
Killip classification	1.476 (1.123–1.940)	**0.005**	1.425 (1.072–1.893)	**0.015**
DM	1.553 (1.018–2.367)	**0.041**	1.590 (1.034–2.446)	**0.035**

OR, odds ratio; CI, confidence interval; RNS, regional network system; DM, diabetes mellitus.

Bold values represent significant *p* values.

### Reperfusion therapy time with regional network systems in patients with and without diabetes mellitus

**FIGURE 2:**
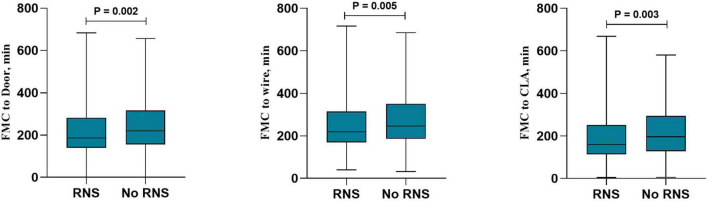
Reperfusion therapy time between RNS and non-RNS in all patients with STEMI. RNS, receive regional network system; NO-RNS, no receive regional network system; FMC-door, the first medical contact to door; FMC-CLA, FMC to catheterization laboratory activity.

**FIGURE 3:**
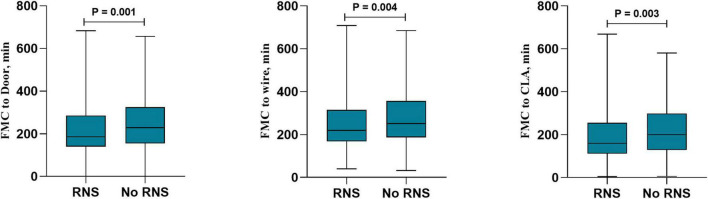
Reperfusion therapy time between RNS and non-RNS in patients without DM and with STEMI. RNS, receive regional network system; NO-RNS, no receive regional network system; FMC-door, the first medical contact to door; FMC-CLA, FMC to catheterization laboratory activity.

**FIGURE 4:**
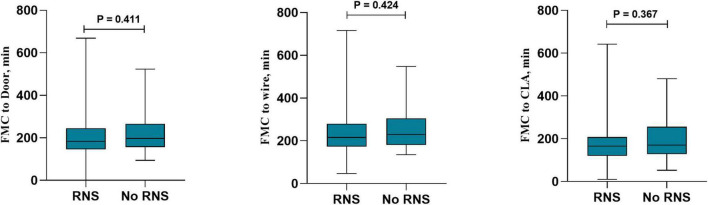
Reperfusion therapy time between RNS and non-RNS in patients with DM and STEMI. RNS, receive regional network system; NO-RNS, no receive regional network system; FMC-door, the first medical contact to door; FMC-CLA, FMC to catheterization laboratory activity.

In the total population, RNS decreased the reperfusion therapy time in all patients with STEMI, including FMC-door [186 (140–282) vs. 220 (154–316), *P* = 0.002], FMC-wire [220 (169–314) vs. 247 (187–352), *P* = 0.005] and FMC-CLA [160 (114–257) vs. 197 (129–295), *P* = 0.003] (Figure 2). RNS also decreased the findings for patients without DM, including FMC-door [188 (140–284) vs. 229 (155–322), *P* = 0.001], FMC-wire [221 (169–315) vs. 255 (188–356), *P* = 0.004] and FMC-CLA [160 (114–257) vs. 201 (133–299), *P* = 0.003] (Figure 3). However, no statistically significant difference in reperfusion therapy time was observed between the No RNS group and RNS group in patients with DM (Figure 4).

## Discussion

We explored the RNS-associated differences in clinical characteristics, clinical outcomes and reperfusion therapy time among patients with STEMI with or without DM. This retrospective study indicated that RNS may decrease reperfusion therapy time in all patients with STEMI and in patients without DM with STEMI; however, no decrease was found in patients with DM with STEMI.

Diabetes mellitus was a major risk factor for cardiovascular disease that may nearly double the risk of AMI (17). DM and impaired glucose tolerance were common in patients with AMI. We identified 148 patients with DM and STEMI, accounting for 14%, a finding approximately consistent with the results of previous studies (18–21). Our study found that patients with DM had more hypertension, higher TG and lower HDL-C than those without DM. These cardiovascular factors may lead to poorer prognosis in patients with DM (22).

Over the past decade, advances in PCI and surgical techniques had greatly improved the treatment of patients with STEMI. The RNS had been demonstrated to effectively decrease reperfusion therapy time in STEMI patients, but the emergence of diabetes may lead to poor prognosis in patients with STEMI. However, it was unclear whether RNS might improve the management and prognosis of patients with DM and STEMI. Our study conducted the first exploration of the differences in reperfusion therapy time in patients with STEMI with or without DM according to the presence or absence of RNS. Logistic regression indicated that DM was associated with receipt of RNS. This indicated that patients with DM were more willing to accept RNS. It was not surprising. DM patients had worse prognosis and complications than no DM and better nursing strategies may be needed in DM patients. Physicians in RNS were familiar with STEMI nursing procedures, and can identify patients’ conditions and provide diagnosis and treatment in time. Our hypothesis was confirmed by serological examination results that patients with STEMI who received RNS had lower troponin, an important risk factor for the prognosis of AMI (23), than those who did not, and this result was observed in both DM and no DM patient. It suggested that RNS may timely diagnose and treat patients with DM, and effectively decrease the risk of poor prognosis to some extent.

Our study found that RNS may decrease reperfusion therapy time in STEMI patients without DM. It was not difficult to explain. Among patients without DM, a higher proportion of remote electrocardiograms were performed in patients with RNS compared with No RNS. Some studies showed that remote ECG can effectively predict the adverse outcome in patients with myocardial infarction and decrease reperfusion therapy time (24–26). RNS was therefore beneficial for the management of patients with STEMI without DM.

However, we found that RNS may not decrease reperfusion therapy time. Possible explanations based on the analyzed results for this finding may be that: (1) In DM patients, there was no difference in remote ECG between RNS group and No RNS group. Remote ECG was a crucial examination to identifying patients with STEMI and DM. One study showed that the incidence of first asymptomatic myocardial infarction and asymptomatic myocardial infarction accounted for 25% of all myocardial infarction in DM patients with no history of atherosclerotic events (27). A study reported that ECG changes in patients with myocardial infarction with DM were not typical, which also caused delays in treatment (28). It suggested that the condition of patients with DM has been suggested to require special management in many aspects beyond ECG. (2) We believe that triglyceride (TG) was also one of the factors. One study found that TG may be an independent risk factor and predictor of DM (29), and another study reported that increasing TG levels within the normal range also led to a sustained increase in the incidence of DM in healthy individuals without metabolic syndrome. These findings indicated that almost everyone may benefit from lower TG. We found that there was no significant difference in TG between RNS and No RNS patients in DM patients. However, RNS group had lower TG in patients without DM, indicating that it was insufficient for RNS in the treatment of blood lipids in DM patients. (3) When patients with DM presented with symptoms of myocardial infarction, their symptoms were often atypical or unusual (30, 31), and their chest pain might not have corresponded to that typical of a heart attack. And it was associated with cardiac autonomic neuropathy, a complication of diabetes, may lead to changes in pain perception (32).

### Limitations

This study had some limitations. First, these data represented relatively small hospital samples and may partially explain the effects of RNS on reperfusion therapy in patients with STEMI with or without DM, but our result found that there was no significant decreasing trend in reperfusion therapy time in patients with DM. A further increase in sample size is necessary for analysis. Second, we might not have considered all relevant confounding factors, and we could not rule out the possibility of reverse causality. Finally, some patients with DM had type 1 DM, but we were unable to perform subgroup analysis to analyze the effects of RNS on the reperfusion therapy time between the two types of diabetes.

## Conclusion

RNS may decrease the reperfusion therapy time in patients with STEMI and without DM. However, we observed no decrease in patients with STEMI and DM, who required enhanced diagnostic procedures and aggressive treatment strategies, including ECG detection and treatment of dyslipidemia. Clinicians must pay attention to prevention strategies, particularly to eliminate modifiable risk factors in patients with AMI and DM. Careful assessment of patients with diabetes and adequate education for the target population are necessary to improve overall survival (33, 34).

## Data availability statement

The original contributions presented in this study are included in the article/supplementary material, further inquiries can be directed to the corresponding author.

## Ethics statement

The studies involving human participants were reviewed and approved by the Medical Ethics Committee of the 920 Hospital of Kunming Medical University (2015067). The patients/participants provided their written informed consent to participate in this study.

## Author contributions

RG was the study guarantor, designed the project, and planned the statistical analysis. XL and LL drafted and revised the manuscript. XL, QY, LY, and LD collected and monitored the data collection. All authors approved the final draft of the manuscript for publication.

## Abbreviations

DM: Diabetes mellitus
STEMI: ST-segment elevation myocardial infarction
RNS: regional network systems
CPC: chest pain Center
FMC: first medical contact
CLA: catheterization laboratory activity
AMI: acute myocardial infarction
ECG: electrocardiography
OGTT: oral glucose tolerance test.
